# Evaluating the effect of a virtual reality digital therapeutic on maternal stress among expectant and postpartum Black and Latina mothers: a protocol paper

**DOI:** 10.3389/fpsyt.2024.1481453

**Published:** 2024-12-09

**Authors:** Mary Carrasco, Stacyca Dimanche, Joshua Fouladian, Marisela Neff, Carolina Scaramutti, Mairead Moloney, Judite Blanc, Azizi Seixas

**Affiliations:** ^1^ Department of Informatics and Health Data Science, University of Miami Miller School of Medicine, Miami, FL, United States; ^2^ Lincoln Memorial University DeBusk College of Osteopathic Medicine, Knoxville, TN, United States; ^3^ Department of Psychiatry and Behavioral Science, University of Miami Miller School of Medicine, Miami, FL, United States

**Keywords:** maternal mental health, virtual reality, pregnancy, postpartum, decentralized trial, digital therapeutic

## Abstract

**Background:**

Maternal mental health is vital to overall well-being, particularly among expectant and postpartum Black and Latina women who experience higher stress levels and mental health challenges. Traditional healthcare systems often fail to meet these needs, underscoring the need for innovative and accessible interventions. NurtureVR, a digital maternal mental health and educational program, leverages virtual reality to offer mindfulness, relaxation, and guided imagery to support these women.

**Objectives:**

This protocol aims to evaluate whether NurtureVR, a digital therapeutic, can reduce stress levels and mental health symptoms in pregnant and postpartum Black and Latina women, improve sleep, enhance pregnancy and childbirth experiences, and increase pregnancy and postpartum health literacy.

**Methods:**

The Nurturing Moms study uses a pre- and post-intervention design with a 5-week NurtureVR program. Fifty participants, 25 expectant and 25 postpartum Black and Latina women, will be recruited through clinics, community organizations, and online platforms. The study involves three phases: baseline assessments of stress, mood, self-efficacy, demographics, and health history; daily use of NurtureVR for 15 minutes during the third trimester and six weeks postpartum; and follow-up assessments at six weeks postpartum. The program includes 49 modules on labor and delivery, stress during pregnancy, nutrition, breastfeeding, hormonal changes, 3D VR representations of fetal development, pain management simulations, and mindfulness exercises for labor. Participants will report stress, anxiety, mood, and pain levels following each VR session. Additional qualitative insights will be gathered through focus groups, and an optional survey will be administered one-year post-intervention to evaluate long-term effects.

**Conclusions:**

The Nurturing Moms study seeks to create a more inclusive and equitable healthcare landscape, demonstrating that digital interventions like NurtureVR are essential for providing high-quality maternal care. By democratizing access to clinical research and healthcare, this decentralized trial promotes equity, improves the generalizability of findings, and accelerates the development of new treatments. The study’s innovative approach has the potential to improve maternal experiences of stress, sleep, and overall health outcomes for Black and Latina mothers and children, despite limitations such as sample size, language barriers, and the preliminary nature of a pilot and feasibility study.

## Introduction

Perinatal stress and other mental health challenges affecting mothers during pregnancy are associated with numerous adverse effects including preterm birth (1). These mental health issues are not limited to pregnancy and may persist or re-emerge in the postpartum period (2, 3). Maternal mental health disparities have been well documented, with expectant Black and Latina mothers at a significantly increased risk of severe maternal morbidity. Black mothers in particular have at least a 3.5 times increased likelihood of maternal death compared to white mothers (4, 5). The high maternal morbidity and mortality can be linked to systemic factors that often affect these populations both prior to and during the perinatal period, with racial/ethnic minority mothers experiencing more discrimination during pregnancy compared to their Non-Hispanic White counterparts (6). Specifically, pregnant Latina and Black women face significant bio-psychosocial barriers and challenges including inconsistent and low-quality prenatal care, lack of education about pregnancy, the birthing process, newborn care, and postpartum challenges. Additionally, Latina and Black mothers may experience limited access to mental health care, financial constraints, social isolation, and systemic discrimination within healthcare settings which can all have adverse effects on the baby and their overall well-being.

Traditional psychotherapy, which serves as the dominant therapeutic intervention, may not adequately address all the needs of these mothers due to its mentalistic and cognitive focus, time constraints, dependency on the therapist-client relationship, and high costs (7, 8). Furthermore, traditional psychotherapy approaches often fail to account for the unique biopsychosocial challenges faced by racial and ethnic minority groups, such as inconsistent prenatal care, lack of education on maternal and newborn health, limited access to quality mental health services, and experiences of systemic discrimination (9–11). As a result, stress and emotional distress among these populations are often not effectively managed. Addressing somatic mental health issues, such as stress, in these populations requires innovative approaches that go beyond traditional psychotherapy to provide more accessible, comprehensive, and culturally sensitive care. One potential, innovative pathway is the use of digital interventions, including virtual reality (VR) interventions. We will detail how the value of VR as a feasible, acceptable, and accessible intervention can potentially bridge the gaps in the unmet care needs of Black and Latina mothers.

The inconsistent and poor treatment of mental health issues among minoritized racial/ethnic groups can be largely attributed to mis- and under-diagnosis of mental health issues (12, 13). There are relatively low rates of mental health counseling for emotional distress in these groups due to stigma and access, but active psychoeducation from a trustworthy source may assist patients and healthcare providers in identifying and addressing perinatal emotional distress to improve maternal and fetal outcomes (14). Traditional therapies for emotional distress in mothers have lacked an educational and preventive component, and the current literature that includes Latina and African-American mothers is limited. However, our team’s own research with expecting Black and Latina mothers suggests that a virtual reality (VR) based therapy can be extremely helpful to mothers from minority backgrounds, especially in the context of addressing perinatal challenges (15).

A recent systematic review of treatments for mental health conditions in the perinatal period demonstrated that psychotherapeutic interventions such as cognitive behavioral therapy and interpersonal psychotherapy were superior to control, but the data is much less concrete in the few studies applying these interventions to low-income or minority populations (16). Similarly, there is limited evidence on the use of psychotropic medications in this population, as low-income and minority patients may have less access to specialty psychiatric care (16). Furthermore, the most recent treatment recommendations for perinatal depression highlight nearly insurmountable barriers to access, including an out-of-pocket cost of $34,000 for Brexanolone and a lack of pregnancy-related data on Zuranolone, both of which have been FDA-approved for postpartum depression in the last five years (17). With inconclusive data on psychotherapy and unrealistic pharmacotherapy options for expecting mothers, it is worthwhile to consider how a novel technology such as VR can improve access to mental health treatments and address gaps in maternal mental health psychoeducation for Latina and African-American mothers.

With a portable and commercially available headset, VR immerses the user into a three-dimensional environment with unique sounds, sights, and images. VR is not only a headset, but also an experience that includes a software program providing a highly detailed 360-degree scene. Its current potential in healthcare includes applications as vast as educating surgeons (18) to restoring executive function in people who have suffered strokes (19). VR has been used in obstetrics and gynecology to reduce pain in laboring mothers (20) and in women undergoing outpatient hysteroscopy (21). VR programs have recently been developed within the community to improve education on hypertension for African Americans (22), and to improve symptoms of anxiety in minority populations (23). Finally, researchers are taking novel steps to reach historically underrepresented groups in research to include them in VR clinical trials (24). Our protocol integrating VR to improve maternal mental health in the perinatal period in the context of a decentralized clinical trial is timely.

Unlike prior studies using VR as a reaction to treat health disturbances, the protocol described in this manuscript, *Nurturing Moms*, is designed as a *preventative* measure for Latina and African-American mothers. NurtureVR, a digital maternal mental health program, developed by BehaVR, was designed to: a) guide mothers through pregnancy via education and empathy and b) help them identify and address potential mental health concerns before these become major barriers to maternal and infant health and well-being. There is minimal research using therapeutic VR for maternal mental health in a diverse group of expecting mothers. Such work is timely, as the scientific community has only recently begun to address the importance of including diverse populations in research (25, 26). This is particularly applicable to digital health research, as the United States healthcare system continues to rapidly integrate technology into patient care. Considering this, researchers and clinicians must make a conscious effort to ensure digital health tools are accessible to all populations, especially those who have been historically neglected by the healthcare system (27).

*The Nurturing Moms* protocol (NurtureVR) addresses the aforementioned gaps in the literature by exploring Latina and Black perinatal mental health and related factors. Our primary objective is to assess whether NurtureVR reduces stress levels and mental health symptoms of pregnant and postpartum Black and Latina women. Our secondary objective is to assess whether NurtureVR improves sleep, as well as pregnancy and childbirth experiences. Our tertiary objective is to examine whether NurtureVR improves pregnancy/postpartum health literacy. We hypothesize that NurtureVR’s combination of mindfulness, relaxation, and education will be protective against postpartum depression, beneficial for pregnancy and birth experiences, and increase health literacy.

## Methods

### Study design overview

*Nurturing Moms* is a pre-and post-design study aimed at reducing stress among expectant and postpartum Black and Latina women through a 5-week exposure to NurtureVR, a virtual reality based digital therapeutic. NurtureVR is a digital maternal mental health and educational program combining mindfulness techniques, relaxation, and virtual reality-guided imagery of pregnant and postpartum experiences. The study was designed to address pregnant women and postpartum mothers experiencing serious psychological symptoms, specifically in the Black and Latina population where mental distress rates are disproportionately higher compared to the general population. Advanced digital solutions can be leveraged to provide quality maternal mental health care that is effective and accessible to all.

The study is driven by three objectives: 1) to assess whether NurtureVR reduces stress levels and mental health symptoms of pregnant and postpartum Black and Latina women; 2) evaluate whether NurtureVR improves sleep, as well as pregnancy and childbirth experiences; and 3) investigate whether NurtureVR improve pregnancy/postpartum health literacy.

### Study procedures

#### Timeline

The Nurturing Moms study will assess the effectiveness of NurtureVR, a digital solution for maternal mental health, across four phases. In phase I, participants will be recruited from health clinics, community-based organizations, and online platforms. Interested individuals will receive a screening form link via REDCap to determine eligibility based on inclusion and exclusion criteria (described below). Eligible participants will be contacted by a study team member to review and electronically sign an informed consent form before completing a 10–20-minute baseline questionnaire on the same platform.

In Phase II, VR headsets will be shipped to participants one week after the baseline questionnaire is completed. Participants will use the NurtureVR for five weeks and will be asked to rate their level of stress, anxiety, mood, and pain level after each VR session using well-validated scales. In Phase III, participants will repeat the surveys three months after completion of the digital intervention. Additionally, two focus group sessions will be held with five participants each, focusing on Black and Latina expectant and postpartum women’s views on resilience, their NurtureVR experiences, perceptions, and recommendations for using VR for stress management. Each focus group will be conducted online to ensure accessibility, with one session scheduled before and one after the participants’ completion of the NurtureVR program. Discussions will follow a semi-structured format, encouraging open dialogue around key themes such as resilience and VR experience. For data analysis, we will employ thematic analysis, using NVivo software to organize and code responses. This approach allows for consistent identification of recurring themes, patterns, and variations in participants’ feedback, facilitating a thorough understanding of their experiences and recommendations. Coding will be iterative and conducted by multiple researchers to enhance reliability and capture diverse perspectives.

**Figure 1:**
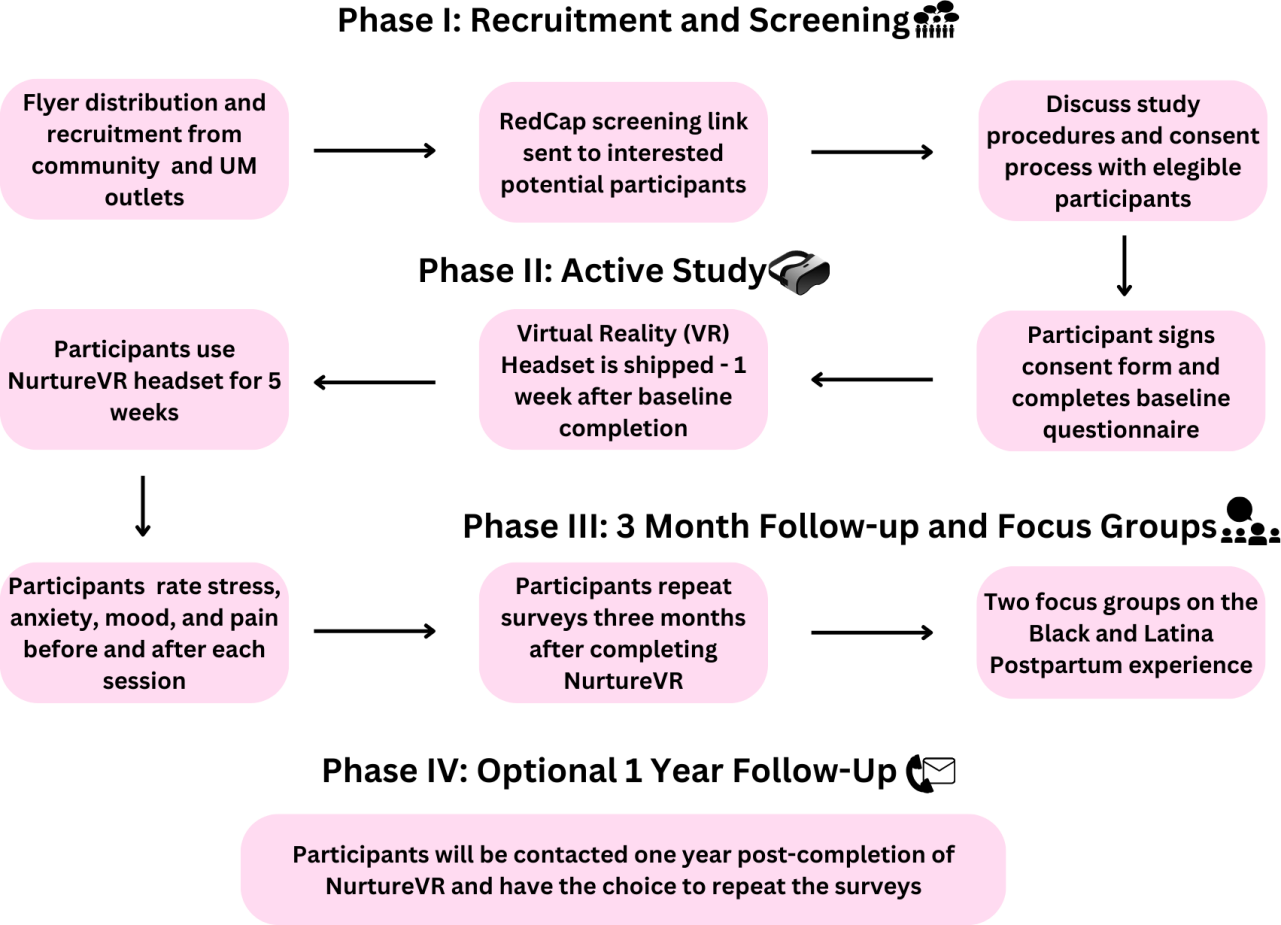
Study timeline.

The final phase, which is optional, invites participants to repeat the surveys one year after completing NurtureVR. This comprehensive approach aims to provide a thorough evaluation of NurtureVR’s impact on maternal mental health in the short and longer term (See **Figure 1**).

#### Participants

Participants in the *Nurturing Moms* study will be recruited through various channels: clinical/health settings, community outreach recruitment events and community organizations, Research Match, and UMiamiHealthResearch.org. Participants will undergo a screening process in which they must meet the following inclusion criteria: 18 years old or older, speaks and understands English, Black or Latina. Additionally, they must be pregnant (less than 36 weeks) or postpartum (up to 12 months after birth). Participants will be disqualified from inclusion in the study based on the following exclusion criteria: a diagnosis of psychosis, inability to speak or understand English, or being neither pregnant (>36 weeks) nor postpartum (up to 12 months). Individuals with a history of seizures, vertigo, significant vision or hearing impairment, significant motion sickness, epilepsy, or sensitivity to flashing light/motion are also excluded from participation. Injuries to the eyes, face, neck, or arms that would make the use of the hardware uncomfortable are also grounds for exclusion. The implementation of these criteria enhances internal validity, optimizing the relevance and reliability of research findings. We aim to enroll 50 participants (25 expectant and 25 postpartum).

#### Study settings

This is a 5-week home-based study primarily using a virtual reality headset to engage in NurtureVR. Participants can set up, customize, and navigate the program through the RealizedCare mobile or web application, which serves as the platform for their participation and is free of cost to the participants. This application is compatible with any cellular device, tablet, or desktop. Mi-Fi devices will be provided to participants who do not have reliable access to Wi-Fi. Participants will be actively encouraged to use the VR headset regularly, by completing at least one module each week (see **Table 1** for module content).

**Table 1:** NurtureVR module content.

Education Topic		Mindfulness Topic
Pre-natal (Before birth)		
Week 1	Power of VR on Mental Health During Pregnancy	Progressive Relaxation sequence
Week 1	Impacts of Acute and Chronic Stress During Pregnancy Techniques to Cope	Turning off Your Stress Response
Week 2	Introduction to Mindfulness and Meditation	Mindfulness: Awareness of Breath
Week 2	Staying Active During Pregnancy	Mindfulness of Body
Week 2	Sleep and Stress During Pregnancy	Relaxing & Letting Go of Thoughts
Week 3	What is a C-Section and Why Would I need it?	Being Present I (Deattachment to Outcomes*)
Week 3	Baby’s Almost Here: Enjoying the Final Weeks of Pregnancy	Mindfulness of the Life Growing Within
Week 3	Baby’s Almost Here: Advantages of Going Full Term	Being Presence II (Deattachment of Outcomes)
Week 4	Stages of Labor	The Healing Power of Breath
Week 4	Pain Management During Labor	Letting Go of Resistance to Sensations
Week 4	Should Hypnotherapy Be in the Delivery Room?	Deepening Awareness
Week 4	What is Lamaze?	Breathing Through the Whole Body
Week 5	Handling Stress and Breastfeeding	Cultivating Connection with Your Baby I
Week 5	Breastfeeding Newborn	Cultivating Connection with Your Baby II
	Lactation Support 2	Honoring Your Body As it Is
All Weeks	Baby This Week module	
Labor (Optional)	How to Use VR During Labor to Reduce Pain	
Postpartum (anyone 6 months after giving birth)		
Week 1	10 Reasons Skin to Skin is Good for Your Baby and You	Cultivating Connection with Your Baby (Redux)
Week 1	Down-There Care: Recovery from Delivery Part 1	Healing is a Powerful Piece of the Journey
Week 2	Postpartum Nutrition	Intuitive Eating/Honoring Your Body’s Needs
Week 2	Sexual Health After Pregnancy	Mindfulness as Empowerment/Acknowledging & Making Choices About Desires
Week 3	Postpartum Emotions	Riding the Waves of Emotion (Staying On Your Raft)
Week 3	How Can Mindfulness Help You and Your Baby?	Sustaining Momentum/Living in the Moment
Week 4	Don’t Let Your Newborn Ruin Your Marriage	Interpersonal Mindfulness (focus on intimacy)
Week 5	Stress Management Techniques After Delivery	Notice Tension/Resistance & Release

#### VR modules

The Virtual Reality (VR) modules designed for perinatal and postpartum mothers offer a comprehensive approach to managing stress, enhancing mindfulness, and providing essential education throughout the pregnancy and postpartum journey. The NurtureVR program was created by Realized Care (previously BehaVR) and they were responsible for the development of the modules.

Nurture VR modules are divided into two main categories: Education Topics and Mindfulness Topics. The Education modules cover crucial subjects, such as the power of VR on mental health during pregnancy, coping with acute and chronic stress, the benefits of staying active, sleep and stress management, understanding C-sections, and preparing for labor. Concurrently, the Mindfulness modules complement the educational content by focusing on practices that promote relaxation, awareness, and emotional well-being, such as progressive relaxation sequences, mindfulness of breath and body, and techniques for turning off the stress response. These VR sessions also address the psychological and emotional aspects of childbirth and postpartum recovery, guiding mothers through mindfulness exercises tailored to cultivate presence, connection with their baby, and overall mental health resilience. This dual approach ensures that mothers are equipped with both the knowledge and the mental tools necessary to navigate the challenges of pregnancy, labor, and the postpartum period with greater ease and confidence. Adherence to the program will be achieved through the Administrative Control Panel (ACP), which is a back-end interface through which registered participants will be organized as well as their module start times, activity time stamps, and completion statuses. Participants will also be asked to fill out mood state surveys before and after completing each module to further ensure adherence. If participants deviate from the protocol, the study team will contact them to address concerns and to remind participants of the study requirements.

#### Recruitment plan

Prior to the recruitment of participants, flyers will be distributed through various in-person or online recruitment channels such as emails to patients’ support/advocate associations, women’s organizations, maternity and parenting forums, maternal support groups, UMiamihealthresearch.org, community outreach events, and offices at the University of Miami Miller School of Medicine Obstetrics and Gynecology department. These flyers will provide an overview of the study, including details on the structure of the intervention, time commitment, eligibility criteria, and compensation. Additionally, the flyer will feature a QR code that interested participants can scan to complete the screening form, along with contact information for the study team to address any questions potential participants may have.

Study team members will host information tables at maternal health clinics, UHealth-associated offices, and various outreach events organized for perinatal women. During these tabling sessions, research staff will introduce the study, guide participants through the study timeline, and demonstrate the use of the VR headset. Interested individuals will have the opportunity to demo the NurtureVR program and complete the screening survey, either on their personal devices or on a study-issued device provided on-site.

**Figure 2:**
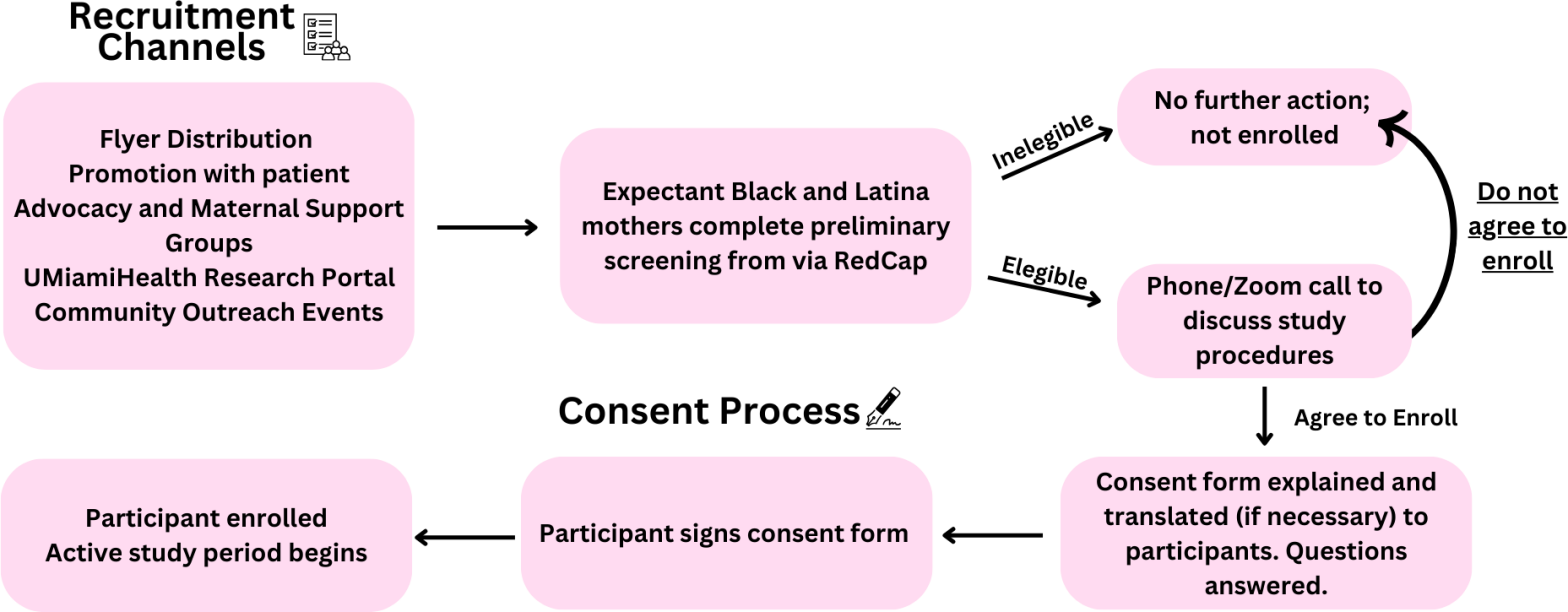
Consent process.

Expectant and postpartum Black and Latina women interested in the study will be instructed to complete a brief screening process to determine eligibility which will be facilitated through a REDCap link provided by study staff. The screening protocol (outlined in the prior section, “Participants”) ensures all participants meet the inclusion and exclusion criteria before enrollment. Participants who meet the established eligibility criteria are contacted via phone or zoom to be informed about study procedures, and asked if they would like to be enrolled and sign the consent form via REDCap to participate (See **Figure 2** for recruitment and consent process). Those deemed ineligible through the screening will be notified of their status by phone or email.

During recruitment, all potential participants will be informed about the compensation details. Participants who complete the 5-week intervention will receive $100 as compensation for their time and effort in participating in the research study. This information will be clearly outlined in the consent form and discussed with participants before taking part of the study.

#### Consent

After eligibility has been established, a copy of the consent form will be sent to the participant via email using REDCap for an e-signature. Interested participants will be contacted to participate in an audio or video call with a study team member. The consent document will be reviewed and explained to the participant at the time of consent, and any questions the participant might have about the study will be answered. Verbal translation will be provided for non-English speakers. Additional questions will be asked by the lab member to confirm comprehension. The participant will be asked if they consent to participate. If the participant agrees, they will be asked to sign and date the consent document electronically. Participants will receive a PDF copy of their signed consent, and a copy will be kept by UM study staff for study records and the regulatory binder.

#### Data and safety monitoring plan

Clinical site monitoring will be conducted to ensure the rights and well-being of human participants are protected, that reported trial data are complete, accurate, and verifiable, and that the conduct of the trial is compliant with the currently approved protocol/amendment(s), with Good Clinical Practices, as well as with applicable regulatory requirements including the University of Miami’s Institutional Review Board.

At the University of Miami (UM), the data manager will manage all study data under the principal investigator’s supervision. The data manager will create computerized data collection forms to ensure the integrity and accuracy of data entry. They will help to minimize the problem of missing data by resolving omissions and errors as they arise. There will be no transfer of data from UM to BehaVR. UM staff will have access to participants’ use data. Safety monitoring will include deliberate assessment and reporting of adverse events to the appropriate Institutional Review Board (IRB) and Privacy Boards. Medical monitoring will also include regular assessment of the quantity and type of serious adverse events. The Nurturing Moms Study was approved by the University of Miami IRB.

#### Statistical analysis plan

For the primary objective, General Linear Model procedures will be used to examine effects of Virtual Reality (VR) on stress over time among pregnant and postpartum moms. For the secondary objective, the User Experience Questionnaire and the Virtual Reality Neuroscience Questionnaire will be used to determine feasibility, usability, and acceptability of NurtureVR as an intervention. Measures of central tendency, and frequency distributions will be used for categorical data.

The main dependent measures will be continuous factors: maternal and pregnancy mental health and sleep scores (see **Table 1** for measures). Regression analysis will be used to explore whether sociodemographic factors (e.g., age, income, education), pregnancy factors (maternal experience scale) and postpartum factors (maternal experience scale and childbirth experience questionnaire) are associated with adverse mental and sleep health outcomes. In the statistical analysis, missing data will be handled using multiple imputation techniques to minimize bias and maintain the integrity of the analysis. Outliers will be identified through visual inspections of data distributions and statistical tests, and decisions about their inclusion will be based on their potential impact on the results and the underlying reasons for their occurrence. We will use sensitivity analyses to assess the robustness of findings. Adjustments for multiple comparisons will be made using the Bonferroni correction or false discovery rate (FDR) control to reduce the risk of Type I errors due to the number of outcomes being measured. Covariates such as age, parity (number of previous births), socioeconomic status, and baseline mental health status will be included in regression models to adjust for potential confounding factors and to provide a clearer understanding of the relationships between the intervention and outcomes. Only factors that show significant correlations in preliminary analysis will be entered in the final model. These data are significant as they will be used to generate effect size estimates (and sample sizes) for planned large-scale studies and to build preliminary exploratory models ascertaining factors that predict mental and sleep health outcomes among Black and Latina pregnant and postpartum women.

#### Ethical considerations

This study protocol and its procedures have been approved by the University of Miami Miller School of Medicine’s Institutional Review Board (study # 20220518). The protocol is in full compliance with the standards set forth by the UM Miller School of Medicine IRB. During the informed consent process, expectant and postpartum moms will be informed on the potential risks and benefits of the research study and have the opportunity to ask the research staff clarifying questions.

### Data collection

#### Baseline and follow-up

After being screened and providing consent for the study, participants will receive an email with a link to complete the baseline survey online via REDCap. This survey includes questionnaires and well-validated scales that collect data on demographics, health history, stress levels, experiences of discrimination, sleep quality, emotional support, and overall pregnancy experience (see **Table 2**). Study team members may help the participant complete the baseline survey over the phone, on Zoom, or in person if necessary.

**Table 2:** Study measures.

Variable*	Baseline	3 months	1 year
Tracking Form and Demographic Questionnaire	x		
Pregnancy Health History or Prenatal Screening Questionnaire (28)	x		
Perceived Stress Scale (29)	x	x	x
Everyday Discrimination Scale (Short Form) (30, 31)	x		
PROMIS^®^ Item Bank v1.0–Sleep Disturbance–Short Form 4a (32)	x	x	x
PROMIS^®^ Item Bank v2.0–Emotional Support–Short Form 4a (33)	x	x	x
Maternal Self-Efficacy Questionnaire (34)	x	x	x
Pregnancy Experience Scale (PES) (35)	x		
Childbirth Experience Questionnaire (CEQ) (36)	x		
Edinburgh Postnatal Depression Scale (EPDS) (37)	x		
User Experience Questionnaire (38)	x		
Virtual Reality Neuroscience Questionnaire (VRNQ) (39)	x		

*Some of these questionnaires have questions about the father. As the father is not relevant and is not a secondary participant in this study, these questions referring to the father have been removed.

**Figure 3:**
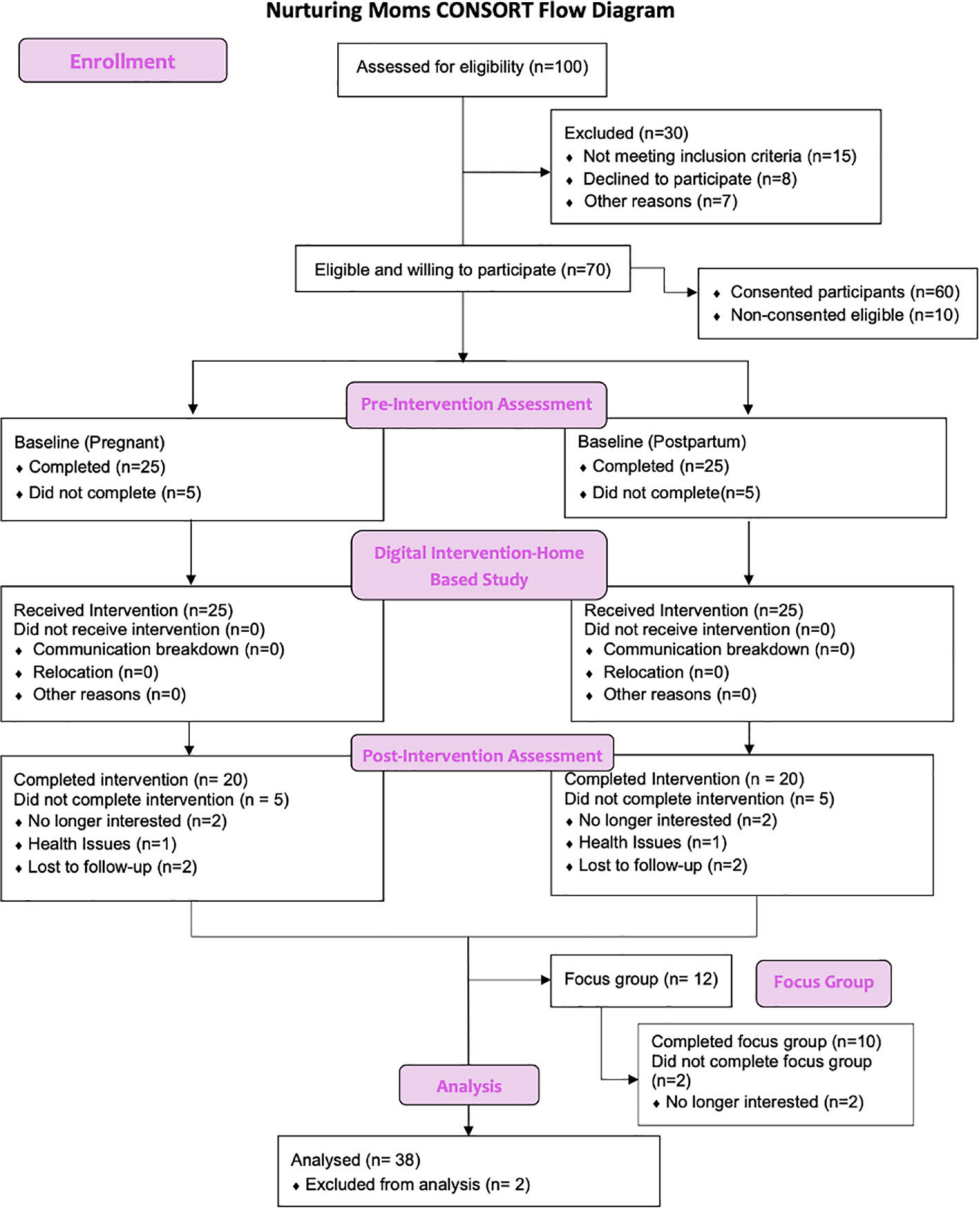
Anticipated recruitment and enrollment for the nurturing moms study.

Participants will be invited to complete the same survey three months following the completion of the intervention and will be monitored for 1 year. There will also be an optional 1-year follow-up (See **Figure 3**).

### Study instruments

1. Tracking Form and Demographic Questionnaire: This form consists of two questionnaires to collect demographic information including age, race, and sex. The forms also capture socio-economic information such as employment, education, income, zip code, and other geographical data.
2. Pregnancy Health History or Prenatal Screening Questionnaire: This 3-page questionnaire developed by the American Medical Association can provide useful material to researchers and physicians who want to gather information either prior to pregnancy or during a pregnancy. This form can help determine the risk of a baby having chromosomal conditions or neural tube defects based on the parents’ family history. The form captures past medical history of the father and mother, mother’s history of pregnancies, and exposures during pregnancy (28).
3. Perceived Stress Scale: The perceived stress scale developed in 1983 is a 10-item questionnaire used to gauge stress levels in individuals aged 12 and older. It examines the degree to which situations are perceived as stressful in terms of how unpredictable, uncontrollable, and overloaded respondents find their lives. Score ranges from 0 to 40, with higher scores representing higher levels of stress. Average scores are calculated by adding up the scores and dividing by the number of items, providing a useful indicator for evaluating the overall level of agreement on the likert scale (where 0 = Never, 1 = Almost never, 2 = Sometimes, 3 = Fairly often, and 4 = Very Often) The perceived stress scale has been shown to have a coefficient alpha reliability of.84,.84, and.86 in three studied samples with a test-retest correlation of.85 (29).
4. Everyday Discrimination Scale (Short Form): The everyday discrimination is a five-item instrument measuring experiences of daily discrimination followed by a question about what the person believes was the reason for that daily discrimination. Responses are coded on a 6-point Likert scale ranging from ‘never ‘= 1 to ‘almost everyday’ = 6. Responses are summed across items to produce a score ranging from 10 to 60 (40). The everyday discrimination scale, developed by David Williams in 1997, has shown high levels of internal consistency, convergent validity and divergent validity among African American men and women, and has been widely used in studies of discrimination and health (30, 31).
5. PROMIS^®^ Item Bank v1.0–Sleep Disturbance–Short Form 4a: The PROMIS Sleep Disturbance and Sleep-Related Impairment item banks were developed using a rigorous and systematic methodology, including literature reviews, qualitative item review, focus groups, cognitive interviewing, and psychometric testing. The short form was developed from the full 27-item bank and has been shown to have convergent validity, construct validity, and reliability similar to the full bank. The short form is an 8-item form measuring the quality of sleep and sleep disturbance over the past 7 days. Four items use an intensity scale (not at all, a little bit, somewhat, quite a bit, very much), three items use a frequency scale (never, rarely, sometimes, often, always), and one item (S109) assessing overall sleep quality uses a scale of very poor, poor, fair, good, very good (32).
6. PROMIS^®^ Item Bank v2.0–Emotional Support–Short Form 4a: The PROMIS Emotional Support Short Form was developed by domain experts from the full bank to capture the range of traits and allow researchers to study secondary outcomes without full additional measures. It consists of 4 statements assessing perceived feelings of being cared for and valued as a person. Responses are coded on a 5-point Likert scale ranging from 1 = never to 5 = always. The convergent validity has been supported by strong correlations between the PROMIS Emotional Support short form and measures of similar constructs. Discriminant validity has also been confirmed by negligible correlations between the emotional support measure and measures of dissimilar constructs (33).
7. Maternal Self-Efficacy Questionnaire: The Maternal Self-Efficacy Questionnaire consists of 10 statements assessing mothers’ confidence in their ability to perform parenting-specific tasks such as understanding the baby’s needs and feelings, talking, reading to and playing with the baby, and taking the baby for regular clinic/doctor check-ups. Responses are coded on a 4-point Likert scale (1 = much worse, 2 = somewhat worse, 3 = as good, 4 = better than others). The questionnaire has been found reliable, face and content validity have been shown to be adequate as well as internal consistency and test–retest reliability for the assessment of maternal self-efficacy (34).
8. Pregnancy Experience Scale (PES): The pregnancy experience scale consists of 10 statements measuring how much different aspects of pregnancy have made the mother feel either uplifted and happy or upset and negative. Each response is rated from 0 = not at all to 3 = a great deal. Internal reliability, test-retest reliability, and convergent validity were comparable to the original 41-item PES developed in 2004 (35).
9. Childbirth Experience Questionnaire (CEQ): The childbirth experience questionnaire consists of 22 statements assessing four domains of the childbirth experience; Own capacity, Professional support, Participation, and Perceived safety. It evaluates women’s experience of labor and childbirth, including their perceptions and feelings. For 19 of the items the response format is a 4-point Likert Scale whereas the last three items use a visual analogue scale (VAS). The scoring range is 1 to 4 where higher ratings reflect more positive experiences. The CEQ has demonstrated internal consistency, discriminant validity, equal item-hypothesized scale correlation, and item equal variance (36).
10. Edinburgh Postnatal Depression Scale (EPDS): The Edinburgh Postnatal Depression Scale developed in 1987 remains a prominent tool for measuring postpartum depression. It consists of 10 statements which identify signs and symptoms of depression in the postnatal period. Responses are scored 0, 1, 2, or 3 according to increased severity of the symptom. Items marked with an asterisk (*) are reverse scored (i.e., 3, 2, 1, and 0). The total score is determined by adding together the scores for each of the 10 items. The EPDS demonstrates acceptable sensitivity, specificity and positive predictive value. It has been used in many studies, and has been introduced as a valuable and powerful postnatal depression screening tool in different cultures (37).
11. User Experience Questionnaire: The User Experience Questionnaire consists of 26-items measuring whether people find the using the NurtureVR product to be an overall positive or negative experience. A seven stage scale is used between two opposing descriptors of the user experience. Mothers rate more closely to the descriptor they identify with (38).
12. Virtual Reality Neuroscience Questionnaire (VRNQ): The Virtual Reality Neuroscience Questionnaire consists of 20-item questionnaire measuring the quality of user experience, game mechanics, and in-game assistance, as well as the intensity of the VR induced symptoms and effects. Responses are coded on a 7-point Likert scale, ranging from 1 = extremely low to 7 = extremely high. The higher scores indicate a more positive outcome (39).

#### NurtureVR

The NutureVR mobile app will be installed on the participants’ mobile device. With 49 modules, the program will span five weeks prenatal, optional use during labor, and five weeks postpartum (available for anyone up to six months after childbirth). These modules are aimed to serve as a sanctuary for learning and stress relief, offering education and support for the entire family while fostering intimate connections and bonding to benefit the well-being of the mother and baby. Key features of NurtureVR include education on the effects of stress during pregnancy, 3D VR representations of fetal development, pain management simulations, mindfulness exercises for labor, and postpartum guidance covering topics such as hormonal changes, nutrition, emotional regulation, breastfeeding, and lactation training through VR-enabled practice sessions.

#### Weekly mood state surveys

**Figure 4:**
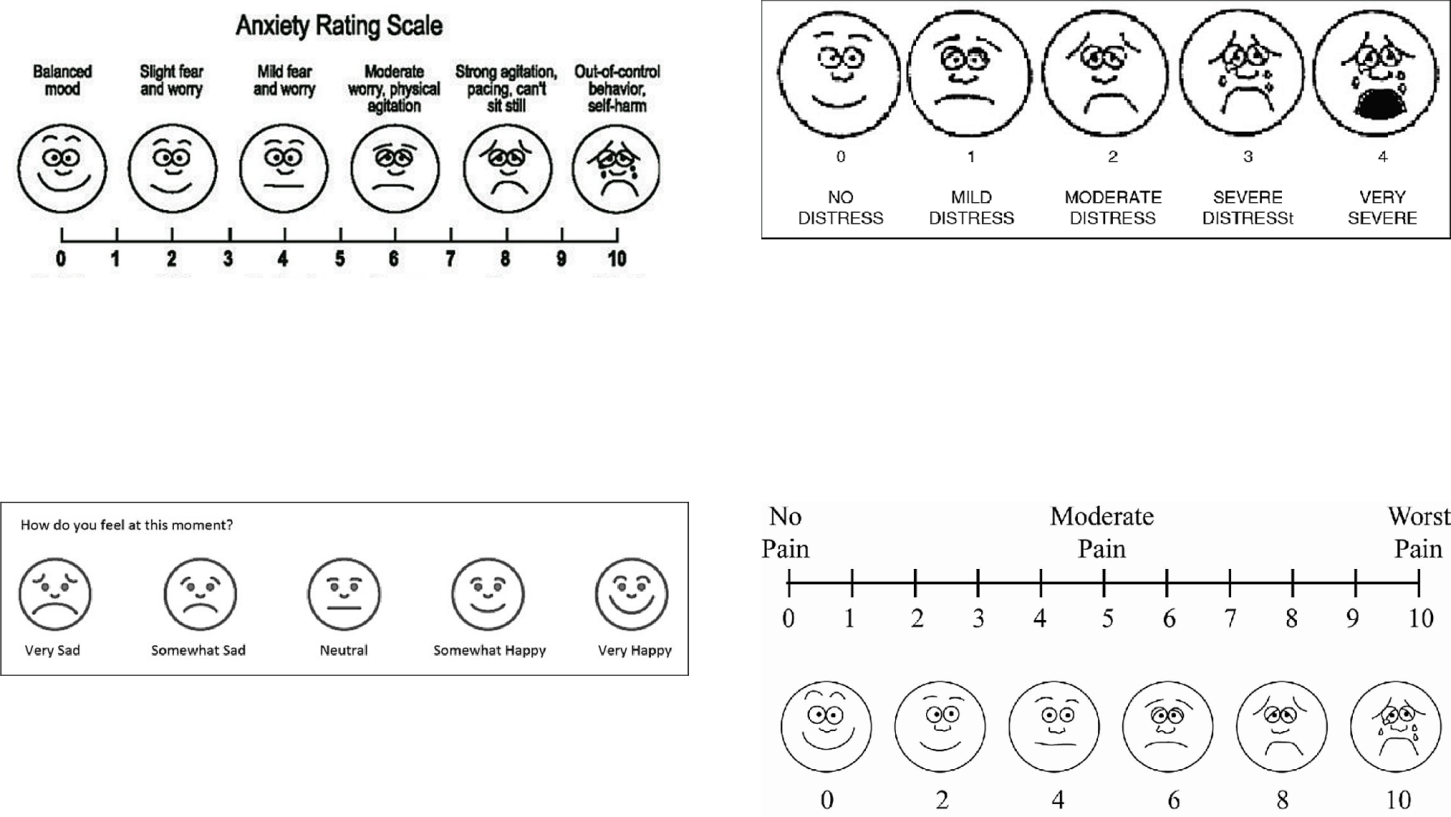
Weekly mood surveys.

Once participants have completed baseline surveys, they will have a maternal health and wellness digital intervention (*NurtureVR* headsets) shipped to their homes. The VR box will include a QR code directly linked to mood state surveys via Google Forms. These surveys consist of four rating scales (see below) that subjectively measure participants’ anxiety, mood, pain, and stress levels before and after usage of the VR (see **Figure 4**). Participants will be asked to scan this QR code after each VR session to complete the surveys. They are encouraged to utilize the headsets as often as they wish, but at least once a week.

1. Anxiety Rating Scale: The anxiety rating scale is a 10-point pictorial Likert scale to measure mother’s feelings of anxiety ranging from 0 = Balanced Mood to 10 = Out-of-control behavior, self-harm (41). Each number corresponds to a face representing the visual level of anxiety. In adults, test-retest reliability of this scale has been used to measure consistency and stability. Analysis using a paired t-test found a significant difference (t(59) = -18.72, *p* <.001) between mean ratings of current and worse pain in the anticipated direction, suggesting that this measure reflects participants’ varying levels of pain (29).
2. Mood Assessment Scale: The mood assessment scale is a 5-item pictorial scale to measure a mother’s overall mood. Each face corresponds to a mood ranging from very sad to very happy. The pictorial mood assessment scale has been shown to have internal consistency and convergent validity in assessing negative affect (42).
3. Pain Scale: The pain scale is a 10-point pictorial Likert scale measuring experience of pain from 0 = no pain to 10 = worst pain. Accompanied by a Visual Pain Analog Scale with 6 pictures of faces corresponding to visual experiences of pain at 0, 2, 4, 6, 8, and 10 of the numerical pain scale. Reliability of the visual pain scale for acute pain measurement as assessed by intraclass correlation coefficients has been shown to be high and sufficiently reliable to assess for acute pain (43).
4. Stress Scale: The stress scale is a 5-item pictorial Likert scale of faces to measure mother’s stress. Each face corresponds to the level of stress ranging from 0 = no distress to 5 = very severe. The stress scale has been shown to have convergent validity in assessing stress and negative affect (42).

## Discussion

The United States is experiencing a maternal mental health crisis. While maternal mental health conditions can affect individuals of all backgrounds, racial and ethnic minorities experience a higher prevalence of pre-and perinatal mental health conditions (e.g., postpartum depression and anxiety), compared to their White counterparts (7). Although perinatal mental health symptoms are prevalent, these conditions are often stigmatized, which makes it difficult for individuals to seek help. One study found that stigma was the most important barrier to women’s help-seeking process (44), limiting them from accessing much needed support and resources. Virtual reality technology is being used progressively to support the treatment of mental health conditions and has several advantages such as convenience and adaptiveness (45). Virtual reality interventions may be one means of offering feasible, acceptable, and accessible mental health care to mothers, who would otherwise fall through the cracks, due to lack of access, or stigma. The scalability of VR technology could enable public health systems to reach diverse populations by integrating culturally adaptive and accessible interventions into existing healthcare services. By addressing common barriers such as stigma and limited access, VR interventions could be implemented in both urban and rural healthcare settings, offering consistent support to at-risk mothers across communities. Furthermore, VR interventions could be incorporated into public health initiatives aimed at preventive care, promoting early screening and treatment for maternal mental health conditions.

This collaboration with BehaVR/Realized Care aims to shed light on the need for more virtual reality-based interventions that promote mindfulness and education, tailored specifically to perinatal mothers of color. By providing mental health support through VR, health providers can normalize the occurrence of perinatal mental health conditions and empower individuals to overcome the stigma associated with mental health, allowing them to access critical services when needed. Such digital interventions offer a unique opportunity to create immersive and engaging therapeutic environments that can be customized to meet the diverse needs of perinatal mothers.

The use of decentralized and democratized digital technology solutions is crucial in increasing access to care. Virtual reality and other digital platforms can bridge gaps in service delivery, making mental health support more readily available to those who may otherwise be marginalized by traditional healthcare systems. These technologies can deliver consistent, high-quality care regardless of geographical or socioeconomic barriers, ensuring that perinatal mothers receive the support they need. By leveraging these innovative solutions, we can foster a more inclusive and equitable healthcare landscape, ultimately improving outcomes for mothers and their children. From a policy perspective, VR could help alleviate strain on mental health services by offering self-directed, immersive therapies that reduce the need for in-person sessions. Public health agencies could integrate VR tools into maternal health programs within clinics, community centers, and even mobile health units, extending the reach of mental health support to mothers in remote or underserved areas. Policymakers could also establish reimbursement mechanisms and funding for VR-based maternal mental health interventions, making them more affordable and encouraging their widespread adoption.

Decentralized trials are essential for advancing clinical research by making participation more accessible and inclusive. *Nurturing Moms*, as a form of decentralized trial, exemplifies this by allowing perinatal mothers to engage in the study from their homes through virtual reality-based interventions. This approach reduces the burden of travel and time constraints, significantly increasing participation rates among underserved and geographically diverse populations who might otherwise be excluded from traditional trial settings. By leveraging digital technologies and remote monitoring, decentralized trials enhance data collection and real-time monitoring, leading to more comprehensive and accurate data.

This model aligns with the *Health at Home* program, which aims to provide high-quality healthcare to all individuals, regardless of their location. The Nurturing Moms study demonstrates the potential of decentralized trials to deliver consistent, high-quality care, ensuring that perinatal mothers receive the support they need in a convenient and accessible manner. By democratizing access to clinical research and healthcare, decentralized trials like Nurturing Moms promote equity, improve the generalizability of findings, and accelerate the development of new treatments and interventions. Ultimately, this approach fosters a more inclusive and equitable healthcare landscape, proving that decentralized trials and programs like Health at Home are essential for providing good quality healthcare for all.

Our proposed study lays out a thorough plan for implementing a mindfulness-based virtual reality intervention among a group of perinatal women of color, focusing on usability, acceptability, and feasibility. Due to the technological nature of this intervention, we anticipate potential technical challenges with the VR headset and with the web-based application. To enhance understanding and encourage weekly engagement with the Virtual Reality solution, we developed specific strategies tailored to common technological issues as well as troubleshooting documentation. Like all research, our proposed protocol has limitations. First, the sample size of 50 participants limits the generalizability of our findings when applied to a broader population beyond the sample being studied. The sample size of 50 participants, divided evenly between expectant and postpartum mothers, was chosen based on the feasibility goals of this study rather than a traditional power analysis. Given the exploratory nature of this research, the primary aim is to assess the feasibility, acceptability, and initial engagement with the NurtureVR intervention rather than to achieve definitive statistical power for detecting clinical effects. The study focuses on understanding user experiences, the practicality of implementing the intervention in real-world settings, and identifying any barriers to using the VR technology among Black and Latina mothers. This approach allows us to gather valuable qualitative insights and user feedback, which will inform the design of larger, more rigorous studies in the future.

The sample size was chosen with a focus on the feasibility goals of this study, while also considering its potential for generating statistically significant results. We conducted a power calculation using G*Power 3.1 to assess whether our sample size could detect meaningful changes in key outcomes. Specifically, we used a t-test to identify means: difference from a constant (one-sample case), assuming an effect size of 0.5, a power of 95%, and an alpha error probability of 0.05. This analysis yielded degrees of freedom (Df) = 44 and a required total sample size of 45. These results suggest that our planned sample size of 50 participants is sufficient to detect significant findings in this exploratory study. While the primary aim is to assess feasibility and acceptability, this sample size ensures that we have enough statistical power to observe meaningful changes in stress levels and mental health symptoms, providing valuable insights to guide future research and larger-scale trials.

The absence of a control group is another limitation that may impact the internal validity of our findings, as it makes it challenging to attribute observed changes in stress and mental health symptoms solely to the VR intervention. The decision to forgo a control group was influenced by ethical considerations, as we aim to ensure that all participants benefit from the intervention, especially given the known disparities in access to maternal mental health support among Black and Latina mothers. To address this limitation, we plan to explore the use of historical controls from similar populations in previous studies and to conduct a randomized controlled trial (RCT) in the future to better isolate the effects of NurtureVR. This future RCT would allow for more robust comparisons and a deeper understanding of the intervention’s efficacy.

Lastly, while the sample design is inclusive of Black and Latina perinatal women, the exclusion of non-English speakers – given that the Nurture VR modules at this time are only provided in English – may also limit the applicability of our results. This may particularly exclude those who do not speak English as their primary language or those with limited English proficiency, especially among Latina mothers. Future research should aim to replicate this study with a larger sample size and develop new modules that are linguistically tailored to different communities. Despite these limitations, the study’s strengths lie in its ability to reach a wider audience of pregnant and postpartum mothers through an educational and mindfulness virtual reality-based intervention compared to traditional methods.

## Conclusion

In conclusion, the *Nurturing Moms* protocol aims to investigate the potential efficacy of NurtureVR, a virtual reality based maternal health and wellness program, in reducing stress among expectant and postpartum Black and Latina women. The disproportionate rates of psychological symptoms among this population, heightened by factors like economic hardships and limited access to psychoeducation, highlight the critical need for customized digital interventions. The present study aims to evaluate the effects of the NurtureVR program not only on maternal stress but also on improving sleep quality and enhancing health literacy during and after pregnancy. Our efforts to maximize the advantages of innovative digital solutions like NurtureVR, which incorporates mindfulness techniques, relaxation, and virtual reality-enabled guided imagery, aligns with recent evidence supporting the effectiveness of psychoeducation and early intervention in improving adverse mental health outcomes among expectant and postpartum women.

The pre-and post-study design provides participants with a holistic approach spanning pregnancy, labor and delivery, and postpartum phases. Implementing comprehensive learning modules, stress-coping mechanisms, and family involvement helps uplift mothers and nurtures the mental well-being of both mother and baby. The Nurturing Moms study not only spotlights the significance of interventions targeting maternal stress among minority populations but also contributes to the growing field of perinatal mental health by leveraging advanced digital solutions to support expectant and postpartum mothers.

## Data Availability

The original contributions presented in the study are included in the article/supplementary material. Further inquiries can be directed to the corresponding author.
